# Physician perspectives on reducing harm and supporting emergency department patients who use drugs

**DOI:** 10.1371/journal.pone.0327899

**Published:** 2025-07-16

**Authors:** Zoe K. D. Collins, Elaine Hyshka, Karine J. Lavergne, Savannah M. Weber, Ginetta Salvalaggio, Cindy Jiaxin Xue, Arlanna Pugh, Janusz Kaczorowski, Aaron M. Orkin, Andrew Kestler, Kathryn A. Dong

**Affiliations:** 1 School of Public Health, University of Alberta, Edmonton, Alberta, Canada; 2 Inner City Health and Wellness Program, University of Alberta, Edmonton, Alberta, Canada; 3 Department of Family Medicine, University of Alberta, Edmonton, Alberta, Canada; 4 Department of Family and Emergency Medicine, Université de Montréal, Montréal, Québec, Canada; 5 Department of Family and Community Medicine, University of Toronto, Toronto, Ontario, Canada; 6 Dalla Lana School of Public Health, University of Toronto, Toronto, Ontario, Canada; 7 Department of Emergency Medicine, St. Joseph’s Health Centre, Toronto, Ontario, Canada; 8 Department of Emergency Medicine, St Paul’s Hospital and University of British Columbia, Vancouver, British Columbia, Canada; 9 Centre for Health Evaluation and Outcomes Sciences, St Paul’s Hospital, Vancouver, British Columbia, Canada; 10 British Columbia Centre on Substance Use, Vancouver, British Columbia, Canada; 11 Department of Emergency Medicine, University of Alberta, Edmonton, Alberta, Canada; University of the West of Scotland, UNITED KINGDOM OF GREAT BRITAIN AND NORTHERN IRELAND

## Abstract

**Background:**

People who use drugs (PWUD) frequently seek care in the emergency department (ED). Little is known about ED physician perspectives and experiences integrating a harm reduction approach into care, including interventions that reduce the health, social and legal consequences of drug use without requiring a reduction in drug use.

**Objective:**

This study aimed to describe the experiences of Canadian emergency physicians caring for PWUD, and facilitators and barriers to implementing harm reduction interventions in the ED.

**Methods:**

Purposive sampling, using an existing national network, and snowball sampling techniques were used to recruit practicing emergency physicians. Semi-structured, one-on-one telephone interviews were conducted until theoretical data saturation was achieved. Interview recordings were transcribed and analyzed using latent content analysis. Interviews took place between June 2019 and February 2020. This work is a secondary analysis specifically focused on harm reduction approaches to care.

**Results:**

32 physician interviews were included. Participants had a median of 10 years of experience (range 1–33) and most (29/32) worked in urban EDs. Participants highlighted the complexities of caring for PWUD, including the intersection of structural vulnerability with substance use. The ED environment varied across Canada and either facilitated or hindered the adoption of harm reduction interventions. Additional barriers included a lack of training and experience; lack of community follow-up care; insufficient ED funding and staffing resources; and, tensions over the appropriate scope of emergency medicine practice. Facilitators included tailored education and training; specialized multidisciplinary teams; ED harm reduction champions; and standardized protocols.

**Conclusions:**

Though variability existed in the adoption and practice of harm reduction in Canadian EDs, most interviewed physicians supported a harm reduction approach to care. To facilitate widespread ED adoption of harm reduction interventions, there is a need for standardized guidance, supplemental resources, facilitated culture change, and sufficient community-based services.

## Introduction

The toxicity of the unregulated drug supply in Canada continues to have a devastating impact on people who use drugs, their families and their communities [1]. Across Canada, opioid toxicity deaths averaged 21 deaths per day from January to September 2024 [1]. The toxic drug crisis is also contributing to increases in emergency department (ED) visits for opioid-related harms [2]. In Ontario and Alberta, opioid related ED visits increased from 12 and 21 visits per day in 2016 to 33 and 43 respectively in 2023 [2]. Across Canadian provinces and territories, the odds of having a repeat opioid-related ED visit (two or more visits in a year) rather than a single visit increased 11% each year from 2016 to 2022 [2].

The ED serves as a critical touchpoint for people who use drugs (PWUD), however PWUD often experience stigmatization and discrimination by healthcare providers [3–9], leading to ED discharges against medical advice [10] and an increased risk of death [11]. These experiences often result in avoiding and delaying future healthcare [4,8,9,12], which heightens the reliance PWUD have on unplanned, urgent care in EDs [12,13]. Ongoing deaths and the devastating impacts of concurrent crises (e.g., lack of housing, infectious disease outbreaks alongside drug poisoning deaths) serve as a call to action for the mobilization of comprehensive services to meet the diverse needs of PWUD [11,14–17].

Harm reduction in health care is a pragmatic, evidence-informed, and patient-centred approach that focuses on reducing the health, social and legal harms experienced by PWUD regardless of whether an individual is seeking to reduce or abstain from drug use [18]. Harm reduction has its roots in a social justice-oriented philosophy that emphasizes community, autonomy, and empowerment [19–23]. Specific harm reduction interventions developed by and for PWUD include overdose response training and naloxone distribution, the provision of sterile syringes and other drug consumption supplies, safer substance use education, and supervised consumption services (SCSs) [5,24,25]. There is evidence supporting the efficacy, feasibility, and cost-effectiveness of these interventions in community [26–29] and hospital settings [30–32], and when harm reduction interventions are combined, substance-related harm is further reduced [6,33]. Over time there has been a push to include some or all of these into traditional healthcare spaces [34,35]. Despite the available evidence, harm reduction interventions, such as syringe distribution programs [28,36] and SCSs [31,37], are seldom available in ED or hospital settings [37].

Harm reduction interventions have sometimes been met with criticism and resistance from some service providers, health system administrators, community groups, and policy makers [38]. This may explain the substantial variability in the application and adoption of harm reduction interventions across Canada [39]. Stigma remains a major barrier to the implementation of evidence-based, patient-centered care for PWUD and has been shown to be deeply engrained within some health care settings [40,41]. Physicians wield considerable influence on decision-making in acute care [42], and as such, physician support is an essential component in changing ED culture and leveraging the necessary ED resources to be able to adopt a harm reduction approach to care.

Understanding emergency physician perspectives on harm reduction as an approach to ED care remains relatively unexplored. The objectives of this study were to examine the experiences of Canadian emergency physicians caring for PWUD, and to determine the facilitators and barriers to incorporating harm reduction interventions in the ED.

## Methods

The present study was embedded within a larger research program led by the Canadian Research Initiative in Substance Matters (CRISM) ED Buprenorphine Working Group, which aims to enhance opioid agonist treatment (OAT) access in EDs [43–46]. The University of Alberta Health Research Ethics Board reviewed and approved our study protocol prior to the commencement of this study (Pro00090060).

This paper offers a secondary qualitative analysis of the original multi-site focused ethnography study, which interviewed Canadian emergency physicians on their perspectives on ED-initiated buprenorphine/naloxone [43], and additionally on harm reduction more generally in the ED. Focused ethnography is a methodological approach that focuses on synthesizing shared practices and beliefs within a distinct sub-group of a community [47–49] and is a suitable method for healthcare research [49] that aims to generate knowledge on a distinct phenomenon to inform clinical decision-making [48,49]. A detailed description of our methods is available within the previously reported primary study [43].

Emergency physicians who had completed their postgraduate medical education training, had at least one year of experience working in an ED, and worked a minimum of four shifts per month in a Canadian ED were eligible to participate. We used purposive and snowball sampling techniques to recruit eligible emergency physicians. The CRISM ED Buprenorphine Working Group national network of ED-site research leads distributed information and recruitment materials to emergency physicians at their respective sites via email. In addition, research participants were asked to share the study recruitment materials with other ED physician colleagues. We set our initial recruitment goal at 30 participants consistent with qualitative sampling for similar research [50].

Our semi-structured interview guide was informed by previously field-tested interview guides for hospital clinicians on substance use interventions and by the lead investigator’s (K.A.D.) experience in emergency and addiction medicine (S1 File). Interview guides were available in French and English, and participants were provided with the choice to complete the interview in either language. Each participant was provided with an information letter and consent form in advance of the interview. One-on-one telephone interviews were audio recorded and began with reviewing the consent form and obtaining recorded, verbal informed consent from each participant. Following each interview, each physician was offered a $50 honorarium to compensate them for their time and expertise. Researchers also maintained field notes where they documented their key perspectives, preliminary interpretations, and overall impressions after each interview. Researchers completed interviews until data saturation was achieved and interviews were not yielding any additional data or themes. Though the interview questions centered on opioid use disorder (OUD) and the initiation of buprenorphine/naloxone in the ED, the interviewers also asked participants about their overall experience caring for PWUD and their perspectives on harm reduction interventions in the ED (e.g., What is your experience caring for people with opioid use disorders in the emergency department? How do you incorporate harm reduction discussions into your ED care, if at all?).

A professional third-party service transcribed interview recordings verbatim and French transcripts were professionally translated to English. Researchers cross-referenced each transcript with its audio recording to ensure accuracy and redacted potentially identifiable information in each transcript. The de-identified transcripts were analyzed using inductive content analysis [51] in NVivo 12 [52]. Data analysis began with data immersion where the primary coder (K.J.L.) reviewed all data, and three secondary coders (A.P., C.J.X., and S.M.W.) reviewed a subset of interview transcripts. All coders discussed their initial impressions and collaboratively developed a coding strategy. K.J.L. then coded all transcripts using an open line-by-line coding technique [48] where sections of the text were coded according to the main topic (e.g., harm reduction, general patient care) and interview content (e.g., harm reduction – facilitator – physician harm reduction philosophy, goal of minimizing harms). K.J.L. and S.M.W. met at regular intervals throughout the coding process to discuss emerging themes and refine the coding tree. A randomly selected subset of six transcripts underwent two quality checks, where S.M.W. confirmed the reliability of the applied codes and C.J.X. confirmed internal consistency of references contained in each code. Strategies for achieving trustworthiness in the data analysis process included data immersion, peer debriefing, negative case analysis, stepwise replication, audit trails, and quality checks [53].

## Results

Thirty-two physicians met the inclusion criteria and participated in interviews between June 21, 2019 and February 11, 2020. As self-reported, participants were predominantly of male gender (n = 19; 60%), between 30–49 years old (n = 25; 78%), worked in urban EDs (n = 29; 91%), and had a median of 10 years of experience (range = 1–33). 13 participants reported working in a high-volume ED (80,00 + visits/year), 13 participants reported working in a low-volume ED (<80,000/year) and 6 participants did not provide the number of ED visits per year. The largest proportion of participants resided in British Columbia (n = 12; 38%), followed by Ontario (n = 7; 22%), Alberta (n = 4; 13%), Nova Scotia (n = 4; 13%), Quebec (n = 2; 6%), Saskatchewan (n = 2; 6%), and New Brunswick (n = 1; 3%). Two interviews were conducted in French and 30 in English. The median interview duration was 56 minutes (range: 36–75 mins). Roughly half (n = 17) the participants described having access to an addiction medicine consult service, either in-person or via teleconference, and 15 participants described regularly providing take-home naloxone kits out of the ED. Where participants were referring specifically to patients with OUD, this was included in the manuscript or in quotes; otherwise, the term PWUD is used.

Results are organized according to our research objectives. The first theme centers on emergency physicians’ experiences caring for PWUD, which describes complexities of care, the perceived need for harm reduction in the ED, and patients’ ability and willingness to engage with care in the ED. The second theme highlights barriers and facilitators to providing harm reduction interventions in ED settings, such as workplace environment and culture, training and education, standardized harm reduction interventions, ED resources, and outpatient follow-up care.

### Participants’ experiences caring for PWUD

#### Complexities of caring for PWUD.

Participants highlighted the complexities of caring for PWUD in comparison to other patient populations. They frequently described care as “*challenging*”; requiring more time and resources than other patient populations and more than what was available in the ED. One of the challenging aspects of care was that PWUD frequently presented with multiple concurrent physical and mental health concerns. Comorbid chronic pain among PWUD was reported as a complicating clinical factor that required additional time and effort. For some participants, they found their typical pain management assessment and treatment strategies were insufficient for PWUD.

*“The patients with opioid use disorders seem to require more, they experience more pain, it’s harder to get their pain under control for specific procedures or for their presenting complaint, just a little bit more challenging. Like, for example, local anesthetic doesn’t seem to work as well.”*

*(Participant 12)*

Some participants also mentioned challenges they and their colleagues faced navigating perceptions of “*drug seeking”* against legitimate acute and chronic pain.

*“So I think what happens is that clinicians and health care providers generally who are working in emergency rooms that are trying to provide care to patients with substance use disorders, is that there is a sort of an assumption that the patient is looking for something, which obviously is not true. I mean, they’re looking for help most times, but there is a perception that they’re going to trick us. […] So that probably is the hardest barrier to overcome in an emergency department.”*

*(Participant 18)*

In addition to multiple comorbidities, participants explained how structural and social determinants of health further increased the time and resources required to effectively care for PWUD. Most participants mentioned the intersection of substance-use related harms and structural vulnerability (e.g., lower socioeconomic status, unstable housing or houselessness, food insecurity, history of incarceration, adverse childhood experiences, experiences of stigmatization/discrimination or trauma in the healthcare system). They indicated that these adverse circumstances complicated care because they can prevent access to follow-up testing, treatment, procedures, and access to wraparound services (e.g., prescription costs, transportation). In addition, the inability for patients to convalesce from acute illnesses in the face of food insecurity, precarious housing, or houselessness also impeded effective discharge planning.

*“P: But just because of the population of people [who use drugs] who end up presenting to emergency, I think that they are disproportionately you know, in tenuous housing situations and frequently don’t have employment or on disability with payments of various kinds.*

*I:Right. And how does that affect the care that they receive in the ED? Or how does that affect your approach or capacity to care for these patients?*

*P:Well, it makes it more difficult, because you’re not discharging them in as safe a circumstance as you might like.”*

*(Participant 11)*

Thus, regardless of a patient’s stated or perceived social stability, interviewed physicians identified that care for PWUD was practically and cognitively more complex than patients who do not use drugs. This complexity limited their ability to systematically offer harm reduction interventions in the ED.

#### Harm Reduction in the ED.

Participants described what their existing harm reduction practice looked like in the context of their ED. They spoke about harm reduction as both an approach to care, as well as a set of interventions. As an approach, they defined harm reduction as part of patient-centered and proactive care, where care is tailored to meet patient-identified needs and circumstances.

“*I think in every patient that we’re trying to help manage substance use, a tailored, customized approach is always required because everyone has their own specific set of circumstances*.”

*(Participant 22)*

A few participants also stressed the importance of supporting patient autonomy.

*“My only caution would be whenever we’re talking intervention and things to do, we have to respect patients’ autonomy. There’s a large subsection of the world who’s using drugs […] and managing and coping and doing quite well, right, in their lives, at least in their framework of what well looks like. And so, you have to give people the autonomy and the freedom to make those choices and so, being willing to have a frank discussion and you know explore whether those are really the optimal choices or the ones they want to make is something that you should be willing and prepared to do. But then you still have to allow people to walk away. The key to this is to be non-judgmental. You know, it’s the, letting people do what works for them.”*

*(Participant 27)*

Participants also described harm reduction interventions they have utilized with the aim of reducing drug-use related harms outside of and within the ED. To reduce drug-use related harms after a patient leaves the ED most participants had experience incorporating at least one harm reduction and/or treatment intervention and a few had experience providing several. Most participants reported they were not—but should be—delivering comprehensive harm reduction interventions routinely to all patients who use drugs. Ideally, these interventions would include: discussions on drug-use related risks (e.g., synergistic effects of drug groups) and safer use strategies (e.g., doing a test dose, not using drugs alone); initiating OAT and/or referring patients to community OAT providers; take-home naloxone kit distribution; offering information on local harm reduction services and addiction treatment programs; and sterile consumption supplies distribution. Participants described practice variation that was contingent upon assumptions about the patient’s social situation wherein there were missed opportunities to provide harm reduction services in the ED to PWUD who have or are assumed to have stable income and housing.

*“I think we sometimes forget the rest of the populations that are still ‘at risk,’ [from drug-use related harms] but who we don’t judge immediately to have [poor] social determinants of health […] The middle-class, upper middle-class patient who comes in after having had some surgery and given a pile of opioids who comes in and still says they have pain and we’re giving them more opioids. But we sort of judged that they wouldn’t be at risk, so we don’t give them protective support, like a naloxone kit or teaching because we’ve sort of separated into ‘them’ and ‘us’ […] Sometimes we miss the opportunity to treat some people who we haven’t flagged as being a concern because of their social determinants.”*

*(Participant 26)*

As well, some participants working in communities with a large network of outpatient harm reduction services explained that they were likely to omit providing or discussing harm reduction interventions in the ED because they assumed that patients were already aware of harm reduction strategies and existing resources.

“*If they’re part of that [inner city] community, chances are they know where the safe injection sites are and how to access them. So [...] I don’t routinely ask people about safe injection site use. Maybe I should*.”

*(Participant 4)*

Most interviewed physicians discussed instances where patients used illegal drugs while in the ED. Apart from the few participants who had access to a nearby or in-hospital supervised consumption service, many participants cited safety concerns for both PWUD (e.g., drug poisoning due to concealed use) as well as staff and other patients (e.g., due to concealed drug consumption supplies). A few participants depicted a recent shift in ED practices away from removing patients who use drugs in the ED from the premises, to adopting a tolerant, non-punitive approach that has improved patient engagement.

*“Once upon a time, if you injected drugs in our washroom or in the hospital, you got thrown out. Now we allow people to inject. If you were to inject in the bathroom, it’s like, “Oh, my God, why did you pass out in the bathroom?” But we don’t throw them out, we treat them and see if they want further information about dealing with their addiction.”*

*(Participant 2)*

Most participants were supportive of the concept of a supervised consumption service available to ED patients but stated that the reality was such a service was not likely to be a funding priority or accepted by hospital or political leadership.

#### Discordance in care goals.

Some participants found that the circumstances under which some PWUD were brought to the ED and the way in which the ED operated led them to question the appropriateness of having prolonged conversations about harm reduction, which they believed may not be desired or understood and retained by patients. In particular, the participants discussed the challenges they faced engaging patients in harm reduction interventions who were brought in via paramedics and treated for an overdose. These patients were described as drowsy, sedated, agitated, and importantly, that “*[T]hey didn’t mean to come to emergency. They weren’t actually actively seeking the help.*” (Participant 31) and therefore were not perceived to be interested in engaging in a conversation about subsequent care. In comparison to PWUD who sought out ED care themselves, this participant explained how for patients who present following a non-fatal overdose, conversations about harm reduction end up “*falling on deaf ears”.*

*“So say maybe someone who’s an IV drug user, and they’re coming in with an abscess, and they’re not acutely high that’s a time when you’re able to have a reasonable conversation. Often [I] find when you’re talking to some of these patients and they just did overdose, or they’re in withdrawal, I feel it’s… everything you’re going to be saying is going to be falling on deaf ears, ‘cause they’re not in the cognitive state for those things. Unfortunately, just because of… the nature of these presentations, it’s not as– there’s not as many ideal situations as I would hope. So I think I don’t do it as frequently as I probably should, but I think it’s really turned me to being a bit of a nihilist. Of thinking, ‘this guy is vomiting, or withdrawing, or is still somnolent or is wanting to get back on the street to get another hit.’ Me having prolonged conversations about harm reduction probably isn’t going to be well-received at this point.”*

*(Participant 1)*

Participants offered concrete strategies for creating a conducive environment to empowering and engaging PWUD in harm reduction interventions in the ED. One key strategy was to have open communication characterized by honesty and trust, the success of which hinged on building good patient-provider rapport. They also stated that engaging family members or peer support workers in patient care created meaningful interactions, where open communication and engagement increased with the encouragement, comfort, and advocacy provided by these supports.

*“I think [peer support workers] would partially address the sense of stigma [...]. Having somebody who has a somewhat shared experience or coming from a similar background, and they understand and are able to predict barriers and sensitivities that might not be otherwise obvious to a healthcare provider.”*

*(Participant 7)*

Participants who did not have access to peer support workers were highly supportive of their role in the ED and anticipated similar benefits. They also mentioned the benefits of ED-based peer support workers would be enhanced if paired with existing community services. By creating continuity with community services, they believed patients would be more willing to engage with peer support workers in the ED and community follow-up care.

### Barriers and facilitators to harm reduction practices in the ED

#### Competing ED priorities and stigma.

Participants highlighted the diversity in ED environments and resources across Canada, stating that this can either facilitate or hinder harm reduction interventions in the ED. Most physicians agreed that a harm reduction approach meant that all ED staff have the shared goal of reducing drug-related harms through patient-centered, empathetic, and trauma-informed care.

However, they stated, the complete adoption of a harm reduction approach in the ED is shaped by several, often competing, influences and can contradict a prevailing ED practice of throughput-oriented treatment of the presenting condition alone. Participants explained how available services can be hindered by political resistance from policymakers and hospital and provincial leadership (i.e., incompatible policies and budgets), communities (i.e., reducing already limited access to healthcare, reluctance to host services in neighbourhood), and staff (i.e., concerns over safety, effect on care of other patients). For example, one physician explained that although they supported the ideas of peer support workers and an in-hospital supervised consumption service in principle, their community’s political realities and constrained resources prevented them from advocating for such services in their ED:

*“We’re a tiny department in a small community with very limited access to almost every staff of health care… In times of just, of austerity, I think we need to have reasonable understanding of the political realities of your community. And I, so no, [an acute care supervised consumption service and peer support workers] would not be something I would advocate for, for right now. And I, that sounds like, horribly conservative to say but I, these are things that I want to see happen, but we are just not, I would not favour allocating those resources to that right now just because of the local blowback we would have for a population who’s already furious that they don’t have access to health care.*

*(Participant 19)*

Further, staff attitudes and stigma can perpetuate an environment that is not conducive to a harm reduction approach to care.

*“These patients are stigmatized and treated differently than the general population […] a lot of the time, they’re physically not let in the main department. They’re kind of left in the main waiting room until they’re waiting to be seen. So I think often there’s a lot of frustration of knowing that […] they’re not worthy of getting a bed or that they’re having to stay outside. At the same time […] they’re quite disruptive to other patients and staff, and rightly or wrongly, the nursing staff here and other places have a pretty low level of patience with them, and they’re often placed in kind of non-care areas so that they don’t become as big a drain on emergency department resources. So I think certainly it’s frustrating and annoying, and probably stigmatizing towards them […] But it’s just kind of the sad reality.”*

*(Participant 1)*

Participants described a perceived cycle of stigma in the ED characterized by staff stigma towards PWUD contributing to negative patient experiences, patient mistrust in care providers, and disruptive patient behaviour in the ED. In turn, disruptive patient behaviour contributed to staff frustration with treating this population and gave way to learned helplessness among staff, which fed back into the cycle of stigma.

*“So, I think that would be again, one of the reasons why staff attitudes could change in the way, in, I think, maybe some of it is as big as transference and counter-transference like, you know if it’s discouraging to keep seeing the same patient and so, you don’t know what else you could do differently. And I also think there’s a feeling of, you know, I guess despair or just, as if you want to give up.”*

*(Participant 16)*

The appropriateness of engaging with patients through a harm reduction approach was also reported as a product of the patient’s physical location in the ED environment. EDs by nature were described as ‘busy’ but also ‘overcrowded’ and not conducive to private, meaningful conversations. Physical space constraints meant that patients may be displaced to hallways. A few participants mentioned that PWUD specifically were more likely to be placed in the hallway. Discussing harm reduction under these circumstances was seen as inappropriate and a breach in confidentiality.

“*We often just put them [PWUD] in a hallway. We’re expected to assess them in the hallway which is extra challenging because it’s often really sensitive topics that you want to discuss but it’s really not appropriate and it breaches their confidentiality to talk about it in the hallway so this really frustrates me.”*

*(Participant 25)*

In addition, some questioned the appropriateness of a harm reduction approach in EDs more broadly, indicating that it was ‘out-of-scope’ and should be primarily addressed in community settings.

*“I think that there’s a risk that the ED is becoming everything to everyone [...] But there’s so many other places that exist in [metro area name redacted] for these other things that I think is probably more appropriate for people to sit down with somebody who can have the time to discuss some of these issues with them. [...] Whether they’re pharmacists, peer support workers, nurses, what have you, social workers, who specifically already have this training and is maybe not being accessed or used to their maximum.”*

*(Participant 5).*

Physicians who perceived this care to be outside the scope of emergency medicine cited the lack of wraparound supports in their ED as a rationale for diverting care to community resources because participants perceived these services to be better equipped to manage the complex needs of PWUD.

Conversely, some participants with more direct experience of harm reduction in their EDs expressed how the implementation of harm reduction interventions preceded practice and culture change; whereby initial staff reluctance to an in-hospital sterile syringe program shifted over time towards favourable attitudes about the program and harm reduction principles.

*I would say it’s getting to be a routine practice. It was, again, hospital policy, it was a struggle for some people to come to terms with the fact that we’re giving needles out for patients to harm themselves but you know, coming around to the idea of harm reduction, it’s better to have a clean needle than a dirty needle, most people are on board I think.*

*(Participant 15)*

To further facilitate the uptake of a harm reduction approach, participants emphasized how having dedicated harm reduction champions in the ED, and sharing patient success stories would increase staff engagement in harm reduction-oriented care.

*“I guess you call that trauma-informed care, whatever. That’s sort of been an epiphany here […] Changing behaviour is the super challenging thing. So, all you can do is offer this smorgasbord of options and hope some people will be receptive […] It would be nice for us to share those stories of people for whom our interactions have actually made a difference […] because it would be helpful for us to have the spirit to continue the work that we do.*

*(Participant 9)*

#### Education and training.

Though emergency physicians felt well trained and prepared to treat acute medical concerns, such as reversing opioid toxicity and treating infections and abscesses, they did not feel that emergency medicine training had adequately prepared them to manage non-acute substance use-related concerns. Many physicians highlighted a desire to learn more about the local substance use epidemiology; opioid prescribing practices, opioid withdrawal, and drug interactions; identifying problematic substance use; available substance use services; and how to reduce stigma and increase compassion for PWUD among ED staff and other physicians.

*“Ways to avoid stigmatizing the patients for both the nursing staff and the physicians. I think that’s one of the biggest issues […] I think that teaching for the medical staff around compassion, empathy, you know, how a person might present. How to potentially identify drug seeking behaviour versus legitimate pain. Not dehumanizing the patients. So I think some training in that regard. I think that the physicians might benefit from some training around how to […] if the decision is made that you might treat someone who has opiate addiction with opiates, how to select the proper one. How to dose it, so that it is effective. You know, things about prescribing if you discharge a patient who has known addiction and you do send them home with an opiate prescription, you know what’s the evidence of that’s going to be, be harmful versus not giving them a prescription.”*

*(Participant 23)*

Many emergency physicians stated that because they are trained in acute care medicine, they often needed to access supplemental training through certification programs, preceptorships with addiction medicine physicians, reading literature, or attending conference lectures, seminars, and/or grand rounds focused on substance use. For participants that had personally sought ancillary training or had ‘hands-on experience’, they stated that this facilitated comprehensive ED care for PWUD. When describing their experience with addiction medicine preceptorships, one participant explained:

*“I didn’t [feel like I had the skills required to care for patients with OUD] until I personally pursued more training in that field. I think I wouldn’t have any idea what I was doing unless I had done, I did, like a preceptorship with the addictions team. Now I work there so every month I’ll do, like, a week there and so that has helped a lot to take those skills back to the emergency department but I think without that formal training it’s tough. Because it is like a different field.”*

*(Participant 14)*

Participants reported a large barrier to the adoption of harm reduction interventions in the ED was staff reluctance towards new harm reduction programs and policies. In many cases, physicians perceived this reluctance to be related to a lack of education and training on the benefits of adopting a harm reduction approach.

*We do have a methadone clinic that is set up in our hospital [...] Just the fact that it is in our hospital […] there are all sorts of rumours circulating “Oh we have a shooting gallery in our hospital”, “Patients are going to rob us, aggress us in the parking lot when we leave after our shift”, […] there would really need to be increased awareness beforehand for the hospital staff […] Perhaps offer some collateral benefits to the ED staff […] then perhaps the nursing staff will see more advantages to this.*

*(Participant 32)*

In depicting their ideals for ancillary training, physicians indicated a desire for education incentives, consisting of professional development credits or financial incentives to cover both training and time away from clinical practice. They also emphasized experiential education, such as preceptorships; short and practical information presented in emails, posters, and lectures; and self-directed, online learning modules.

#### Standardized practices.

Participants observed substantial variability in the delivery of harm reduction interventions in the ED and highlighted how standardized protocols and guidelines have previously facilitated the adoption of new practices in the ED, such as the distribution of take-home naloxone kits. However, they also expressed a desire for the development and implementation of materials (i.e., protocols, guidelines, consensus statements, best practices) that would more broadly standardize care of PWUD in the ED, thus ensuring that every PWUD who seeks ED care also receives the offer of comprehensive substance use care.

*Patients come in with the same complaint on different days, they should get something resembling the same care and unfortunately this is sort of one of the highest, right now, the highest practice variance pieces of our group and so, you know, it’s sort of, was chief, I guess chief of our group I would really like to get some other support so we can improve and minimize our variance […] I sometimes wonder whether it would be better to just, to not, to not do it at all because so it’s so suboptimal. But at the same time, and primarily my concern is to the [regulatory] college and that our practice environment is such that, you know, there’s this sort of focus on people shouldn’t be prescribing opioids out of the emergency department and yet, there’s no other option… I think there are times where that does put some of our patients at risk [of withdrawal] and it, I would like to see sort of better, better pathways.”*

*(Participant 19)*

These resources would include guidance for physicians and ED staff on the delivery of harm reduction interventions, how to manage certain patient behaviours (e.g., using opioids in undesignated spaces), and opioid prescribing practices related to pain and withdrawal management.

#### ED resources.

Participants described the facilitating impacts of having specialized, multidisciplinary care teams and dedicated general ED staff with experience caring for PWUD and knowledge of harm reduction and community resources. Multidisciplinary teams or centralized outpatient addiction services in the hospital were perceived as key strategies to provide essential wraparound social and mental health supports resulting in comprehensive and quality care for PWUD, rather than ‘piece-meal’ harm reduction interventions. In addition, participants advocated for access to reliable in-person or virtual addiction consultation services to facilitate the delivery of harm reduction interventions and addiction treatment in the ED. Though participants stated that consults with addiction services or clinicians with addiction medicine training enabled high-quality ED care for PWUD, a lack of evening or weekend coverage created a barrier to care during ‘non-conventional hours.’

*“There’s a physician-based consult service as well as an addiction nurse specialist [...] outside of the hours of maybe like 11pm ‘til 7am, there’s someone there who can see the patient while they’re there. It’s quite amazing.”*

*(Participant 22).*

Despite identifying expert consultations, multidisciplinary teams, and experienced staff as the ideal environment, participants recognized how the implementation of such services is constrained by the hospital’s fiscal responsibilities and available human resources.

*“In a perfect world, I think it would be a very integrative, collaborative, inter-professional group that includes physicians, nurses, social workers, pharmacists, peer support workers, allied health, psychologists, psychotherapists, so on and so forth, that can address the full spectrum and the full physical and mental health needs of individuals who have this problem. But like I said, all of those things require money and resources which I think are tight, right? It’s bringing them all together in one unit that in a perfect world, again, would be operating 24 hours a day, 7 days a week, 365 days a year.”*

*(Participant 20)*

In many interviews, participants indicated that a lack of space, funding, and staff prevented the full implementation of harm reduction interventions into ED care and advocated for more funding to increase ED capacity to support PWUD. Additional funding could enable modifications to ED infrastructure, such as changes to washrooms to improve the safety (e.g., adding call bells and alarms, adding three-quarter length doors to stalls). Upon discussing the addition of supervised consumption services in the ED, physicians described the need for additional funding to create a dedicated space and to hire staff. Without this funding in place, one physician described how the potential increase in workload would hinder buy-in from current ED staff:

*“The big issue for us is obviously funding, if they could provide us with a dedicated nurse, and a dedicated space then, and dedicated security then I think [a supervised consumption service within the ED is] something that would certainly be worth discussing. I think that again, it’s the idea of an added work for the emergency department, is always the fear for many of my colleagues.”*

*(Participant 13)*

Participants’ perspectives on essential harm reduction resources were often determined by the perceived demand for addiction services in the ED as well as the availability of local community resources. Although most participants held favourable attitudes toward supervised consumption services, sterile syringe distribution programs, take-home naloxone programs, and peer support workers, many indicated that their EDs do not offer these services.

*“It’s a function of how big you are, like I would love to have supervised consumption booths in our [ED] and on the inpatient ward would be amazing but it’s not really realistic for our volume here, we just don’t have that many patients. In you know, in [a larger urban centre], absolutely you should have it, and if I was working there I’d be pushing it too.”*

*(Participant 15)*

Participants who worked in rural settings, with limited ED and community harm reduction services described how enhancing access to primary care, and the capacity of generalist providers would be a more effective and practical strategy to improve care for PWUD.

*Like I think that, that’s my concern with some of this stuff that this sort of like, high level urban focused practice opioid use stuff, what my patients that we’re taking care of with this need is, is someone with a general skill set and a degree of specialized knowledge and that, and to have sort of more of that generalist thrust, or generalist contribution to the research, I think that’s really important.[…] And it’s particularly important in the rural populations that, at least where I live, just have terrible access to primary care*.

*(Participant 19)*

As such, participants stressed the importance of having harm reduction resources in the ED that are tailored to the local context.

#### Community follow-up care.

There was consensus among participants that timely, reliable, and low-barrier access to community harm reduction, mental health, addiction treatment, and social support follow-up services facilitated ED care for PWUD. However, many participants reported being unfamiliar with the local resource landscape and were particularly concerned with improving care transitions to minimize loss to follow-up. To circumvent loss to follow-up, participants suggested establishing integrated care pathways and referral protocols to community resources; creating formalized community partnerships; and having specialized addiction human resources or experienced support staff (e.g., nurse, social worker) to help coordinate follow-up care and linkage to community resources from the ED.

*“What would really help in general is having, whatever you want the title to be, but somebody ideally from a social work background who is able to be like a patient navigator […] so, we could start medical therapy in the ED, especially for opioid use disorder, but getting them in contact with all of the services that may be available to them getting them hooked up with detox, rehab, etcetera, that’s all way beyond what we have the time for in ED or honestly the expertise for. So, a patient navigator that’s maybe on call or just someone that they could get in touch with the next business day would be great. And we don’t have that.”*

*(Participant 15)*

Participants who described having access to centralized community intake and referral services, integrated outpatient addiction clinics, or who had established formal partnerships with community prescribers (e.g., opioid agonist treatment clinics, pharmacists) reported being satisfied with the accessibility of follow-up care in their communities. However, other participants, particularly those in rural areas, noted the absence of, or inadequate access (e.g., limited capacity) to, follow-up care as a large barrier to providing comprehensive care in the ED.

## Discussion

Our study revealed that participants perceived care for PWUD as complex and multifaceted given a higher proportion of comorbid health and social concerns, substantial variability in patients’ ability to engage in care, and a greater need for wrap-around medical and social supports in comparison to other patient populations. Participants spoke of the importance of providing patient-centered, proactive, non-judgemental, multidisciplinary, and empowering care that supported patient autonomy. They were generally supportive of integrating harm reduction interventions such as syringe distribution and supervised consumption services into ED-based care, however, cited barriers related to ED resource constraints and cautioned that the ED must also be part of a continuum of care for patients, that is tailored to the local context, and provides robust access to adequately resourced health and social services in the community.

While emergency physician perspectives on ED harm reduction services have not been widely examined previously, our findings were similar to what has been previously reported for other ED-based interventions for PWUD such as ED initiated buprenorphine/naloxone [54–58] including the findings on OAT access in Canadian EDs reported in prior analyses from this study’s overarching research program [43,46]. Analogous to our findings, emergency physicians in Canada [43,46,58] and the US [54–57] have described similar barriers to ED-initiated buprenorphine/naloxone. These barriers included a lack of training, education, and experience [46,54–56]; availability of community follow-up care [46,55,57,58]; insufficient ED funding and staffing resources [46,54]; limited time, physical space, and competing priorities [55–58]; beliefs about scope of practice for emergency medicine in OUD management [43,54,58,59]; lack of patient engagement [43,57,58]; and complexity of care [43,58]. Facilitators to care have included tailored education and training [43,55,57]; multidisciplinary teams [57]; expert consultations [43,46,57]; ED champions [58]; sharing success stories [54]; peer counselors [57]; and having standardized protocols, recommendations, or order sets [43,55–58].

Two recent studies examining harm reduction in the ED from a systems perspective—one a case study and the other a mixed-method study involving surveys and interviews with ED nurses and physicians— reported comparable findings, suggesting consistent experiences integrating harm reduction in the ED across healthcare provider roles [60,61]. Specifically, both studies identified an ED mandate that prioritizes patient throughput over relational and harm reduction oriented care, a lack of harm reduction education, and constrained staffing resources [60,61]. By applying a systems lens, these studies highlighted how individual factors interact to shape harm reduction efforts in the ED [60,61]. They found that organizational policies, team dynamics across different roles (e.g., nurses, physicians, and addiction medicine specialists), and interactions between specialists and non-specialists can either enable or constrain harm reduction practices [60,61]. These findings reinforce the need to consider how barriers and facilitators interact in complex ways and vary across contexts. Recognizing these relationships should inform the development of standardized harm reduction recommendations and implementation guidance.

National guidelines focused on the treatment of patients with OUD [26,62], while recommending harm reduction services, lack implementation recommendations specific to acute treatment settings. The Canadian Association of Emergency Physicians (CAEP) has published a position statement on the management of people with OUD in Canadian EDs [63]. While this statement specifically states that EDs should provide sterile drug consumption supplies, overdose prevention education, take-home naloxone kits, and referral to local overdose prevention sites and/or supervised consumption services, it lacks accompanying documents such as standardized protocols to support implementation. The development of such tools and extension of existing guidelines to include care in ED and acute care settings could support implementation. For example, Bridge, a program of the California Public Health Institute, has developed a guide for implementing harm reduction in the emergency department which could be adapted for Canadian contexts [64]. Further, the National Institute on Drug Abuse Center for the Clinical Trials Network’s quality framework for the treatment of OUD in US ED settings offers concrete structural, process, and outcome measures [17], with recommendations aligned with the guidance provided by participants in our study.

To further align these standardized protocols on caring for PWUD in EDs with the findings in this study, they must include some flexibility to adapt to local needs, specifically, ED patient demand [54] and access to community follow-up care. EDs could potentially benefit from conducting a patient needs assessment and environmental scan of local resources before deciding which harm reduction interventions to offer. Policymakers and hospital leaders can support the implementation of harm reduction interventions in the ED by prioritizing implementation evaluations as well as earmarking funds and resources for such activities. Hospital and ED leaders may also want to share the results of these assessments with their staff and physicians as a form of education on the existing resources available in their communities and to demonstrate merit in providing harm reduction interventions in the ED. Ancillary training could also reduce stigma and increase physicians’ confidence in treating PWUD, which has been shown as a barrier to emergency physicians providing harm reduction interventions in our study and in the literature [56,58]. Hospital and ED leaders can support ancillary training with incentivized addiction clinic preceptorships or shadowing addiction consult clinicians in their own or a neighbouring ED.

Though our study found that all participants had some experience incorporating harm reduction interventions into their clinical practice, many reported that they did not — but should — offer such interventions routinely and comprehensively. Variability in emergency physicians’ attitudes and practices have been reported previously [46,57,58] and one study cited underlying stigma as a cause [46]. Patel and colleagues’ [54] qualitative study explored staff perspectives on buprenorphine/naloxone initiation and ‘warm handoff protocols’ in Pennsylvania hospitals and indicated that effective implementation of ED OUD care is contingent upon addressing staff stigma and bias to increase buy-in [54]. Conversely, some participants in our study indicated that harm reduction policies can, over time, reduce the ED culture of stigma surrounding caring for PWUD – revealing that policy implementation could precede initiatives to reduce the stigmatization. Adopting a more patient-centered and trauma-informed approach, alongside offering evidence-informed harm reduction interventions, may help alleviate frustrations stemming from misperceptions that patients are unwilling to engage in care, and reduce stigma. This could be achieved by co-designing care models in partnership with PWUD to ensure care is responsive to both patient needs and ED realities. This approach could enhance patient care and the mitigate the moral distress that arises when physicians are caught between a high-throughput ED mandate with insufficient resources, and a duty of care that goes beyond patient’s immediate needs [65,66]. ED-based champions, institutional support, standardized order sets and education can be effective starting points to change the way care is provided [67]. Additionally, hospital-based supervised consumption services are a promising evidence-based intervention that may reduce the risks of in-hospital drug use and transform hospital culture, as endorsed by participants in this study and healthcare providers in British Columbia [60] and Toronto [68]. Aligning the ED care environment with patient needs and provider abilities can help address moral distress, practitioner burnout, compassion fatigue and vicarious trauma – breaking the cycle of stigma towards patients who use drugs in the ED (pg.e13) [40].

In the ED, peer support workers and addiction medicine consult services are potential specialized supports that can reduce the burden on emergency physicians and staff but require additional resources [69]. Peer support workers can promote active patient involvement in care planning and facilitate connection to addiction treatment and/or recovery services [70]. Addiction medicine consult services can assist with the care of patients that require more complex addiction treatment initiation, wraparound health and social services, health system navigation, and can support formal and informal education of ED staff. For busy, high volume EDs that serve a large population of PWUD, the addition of in-hospital addiction medicine consult teams could support ED physicians’ educational needs, assist with complex patient care, champion the implementation of new services and act as local or regional experts to support implementation at other sites. In EDs that do not identify a need for an on-site addiction medicine team, provincial or regional virtual referral services [71], as well as the development of local partnerships with clinics, pharmacists, and other community prescribers (e.g., primary care physicians with crossover addiction medicine training) may be a means to support emergency physicians caring for PWUD and ensure patient access to outpatient follow-up services. While additional resources and facilitated generalist-specialist collaboration are key, EDs must commit to adequate training, care standardization and reducing stigma within their own environments.

Beyond adding specialized staff and resources to assist with the care of PWUD, EDs should consider the overarching principles of harm reduction, including its roots as a social justice movement, to explore other ways of addressing the structural and social contributors to substance use harms. The successful adoption of a harm reduction approach requires a shift away from addressing the immediate harms of substance use to addressing the root structures that predispose PWUD to harm [5,25,72]. A structural vulnerability lens can help explain the inter-related, dynamic, and mutually reinforcing factors that shape the adoption of harm reduction in ED settings, patient care interactions, and substance use-related health outcomes. The structural vulnerability framework posits that there are distinct patterns of experiences and health outcomes among individuals and groups based on their social identity (e.g., race/ethnicity, gender, class, sexuality, ability, legal status, risk environment) interacting with hierarchical networks of power (e.g., political, institutional, economic, cultural/normative, social structures) and their embodied effects (e.g., compounding stigmatization, discrimination) [3,5,7,73–77]. The resulting privilege or oppression is magnified by socially imposed attributions and assumptions (e.g., health deservingness) that become embodied within the individual or group’s behaviours, which further enables or constrains vulnerability to and experiences of suffering and harms [73,76,78]. The complexity-informed and intersectional nature of the structural vulnerability framework is compatible with the harm reduction movement and can help disentangle the structures that shape the adoption of harm reduction in Canadian ED settings, as well as identify possible points of intervention to improve equitable care for PWUD. One approach would be the implementation of a standardized assessment of structural vulnerability [73], alongside additional training [79], to help identify patients who are likely to benefit from additional health and social services. This is in alignment with the Canadian Medical Association Code of Ethics and Professionalism which states, “Recognize that social determinants of health, the environment, and other fundamental considerations that extend beyond medical practice and health systems are important factors that affect the health of the patient and of populations.” [80]

### Limitations

This study completed interviews in early February 2020, prior to the first wave of COVID-19 in Canada. The onset of COVID-19 changed the ED landscape dramatically as resources were diverted to respond to the pandemic despite increasing rates of opioid-related ED presentations [81]. As such, the results of this study may not be representative of current emergency physicians’ perspectives. Physicians may have shifted focus away from providing harm reduction interventions, or conversely, demand for these interventions may have increased since the study and physicians focused more on developing these practices.

Inherent limitations in qualitative research include response bias. Though researchers cannot control how their presence may influence participant answers in interviews, the four interviewers were trained to avoid non-neutral prompting to reduce the risk of response bias. Further, data immersion, peer debriefing, negative case analysis, stepwise replication, audit trails, and quality checks were completed as part of the data analysis processes.

Selection bias may have occurred through our recruitment strategy when emergency physicians self-selected to participate and may have recruited additional participants with similar views with snowball sampling. To mitigate these limitations, we encouraged site leads to recruit physicians with diverse perspectives. However, as a result, our sample may not be representative of all Canadian emergency physicians, hence why this study was part of a multi-method program of research [43,45,46]. Further, this study explored the perspectives of emergency physicians and omitted the perspectives of other ED staff. Multidisciplinary healthcare teams and the interactions between healthcare provider roles appears to have a critical influence on the implementation of harm reduction in ED yet the perspectives of specific staff roles is underexplored in the literature [60,61]. Future research should further examine the perspective of diverse emergency department staff to better understand how different roles conceptualize and integrate harm reduction into ED patient care.

Lastly, this study did not include the perspectives of patients. Advancements in harm reduction are most responsive and inclusive when people with lived experience of drug use, and service providers, are directly involved [82]. Future research on the design, implementation, and evaluation of harm reduction approaches in the ED should directly engage PWUD as partners.

## Conclusion

Though variability existed in the routine application of comprehensive harm reduction interventions in Canadian EDs, most interviewed physicians supported this approach to care. To facilitate the wide-spread adoption of harm-reduction interventions within ED care, physicians indicated a need for standardized guidance in caring for PWUD with enough flexibility to adapt to local needs; supplemental resources to increase the ED’s capacity for harm reduction without impacting the ED’s ability to provide services for other patients; facilitated culture change through policy implementation and education; and, adequate community-based addiction treatment, harm reduction, and social supports to ensure a successful transition from ED to community-based care.

## Supporting information

S1 File Semi-structured interview guide. (PDF)
