# Supplementary material for: Physician perspectives on reducing harm and supporting emergency department patients who use drugs
Source: PLoS One. 2025 Jul 16;20(7):e0327899. doi: 10.1371/journal.pone.0327899 (PMC12266462; doi:10.1371/journal.pone.0327899)
Supplement: S1 File — (PDF) [file pone.0327899.s001.pdf]

S1: Physician Qualitative Interview Guide

Emergency Department Physician Attitudes Towards Buprenorphine Initiation in the ED

[Introduce myself: Good morning/afternoon, my name is XX, and I work with the Inner City Health and Wellness Program in Edmonton. We are doing a study in partnership with the Canadian Research Initiative in Substance Misuse.]

The purpose of this interview is to get your perspective on the role of the emergency department and the emergency physician in caring for patients with opioid use disorders. We would like to hear your opinions on initiation of opioid agonist treatments like buprenorphine/naloxone in the ED, how the ED can best care for patients with opioid use disorders and how caring for these patients has impacted front line staff. As a reminder, your participation in this study is voluntary, and you may skip any questions you don't want to answer or stop the interview at any time without penalty. I will audio-record this interview, which will then be transcribed verbatim. Any information that may identify you will be removed from the transcript prior to analysis. We may use direct quotations from you in the reported findings, but these quotations will never be linked to your name; rather, we will use a generic description of your professional role to provide context for your comments.

[TURN ON AUDIO RECORDER]

Pre-Interview: Demographics and Education

**Questions:**	
**We would like to collect a few demographic identifiers before we start the interview. Can you tell us your:**	- First and Last Name<br>- Gender<br>- Telephone Number<br>- Email Address<br>- Current Age<br>- Urban vs. rural

	- Number of visits per year (approximately)
**We would like to collect information on your education history and current place of employment. Can you tell us: How long have you worked in the emergency department?**	- Did you train in Canada or abroad? Where?<br>- How many years of training post-medical school did you complete?<br>What types of patients do you typically see (special populations, including pediatric patients, young adult patients)<br>- Do you have any other areas of practice?

Topic Area I: Caring for patients with an opioid use disorder

**Questions:**	**Possible probes:**
**What is your experience with caring for patients with substance use disorders?**	- What changes have you seen over time with respect to patients seeking care for substance use related issues?
**What is your experience caring for people with opioid use disorders in the emergency department?**	- Specific examples/incidents<br>- Frequency of encounters<br>- Positives or negatives<br>- How does caring for these patients differ from other patients who don’t use substances, if at all?

	- How does caring for these patients differ from other patients with substance use disorders (e.g. using stimulants, alcohol, etc.), if at all?<br>- How are youth with opioid use disorders managed at your site?
**Sometimes social determinants of health, such as race, gender and class, can influence how people with opioid use disorders are perceived and treated in the ED. What has been your experience of this at your site?**	- Any specific examples featuring differences in care based on <u>race</u>?<br>- Any specific examples featuring differences in care based on <u>gender</u>?<br>- Any specific examples featuring differences in care based on <u>class</u>?
**Do you think that your emergency department does a good job caring for patients with opioid use disorders?**	- Are there things that we should or should not be doing?<br>- Are there things that place patients at risk?
**Do you feel you have the skills required to take care of patients with opioid use disorders in your ED?**	- What skills are you missing?<br>- How should training be provided to ED physicians?<br>- Have you taken any extra training in this area?
**Do you have timely access to an addiction medicine consult service, phone advice line or another way to**	- If not, would this be helpful?<br>- What else could make your job easier (including designated care

**access expert advice for patients with opioid use disorders?**	spaces, specialized human resources such as addiction nurses and peer navigators)?<br>- What is good / bad about these services?
**We've heard from patients that they sometimes feel stigmatized in the ED. Why do you think that is?**	- Injection drug use, social factors (e.g. homelessness), race?<br>- Do you have any strategies in your emergency department to address this?

Topic Area II: Initiation of opioid agonist treatment in the ED

Questions:	Possible probes:
**Do you have a way to systematically identify or screen patients for high-risk opioid use in your emergency department?**	- If yes, how so?<br>- If no, would something like this be helpful?
**Have you ever started a patient on buprenorphine/naloxone in the ED?**	- How did you find this experience?<br>- What made it easy or difficult?<br>- If no, would you feel comfortable doing this on your next shift?
**What are the main barriers to initiating buprenorphine/naloxone in the ED?**	- Is the risk of precipitated withdrawal a barrier (why or why not)?<br>- How do you treat/manage precipitated withdrawal?

**Do you think that your ED is an appropriate place to initiate patients on buprenorphine/naloxone treatment for their opioid use disorder?**	- Why or why not?<br>- Where is the most appropriate place?<br>- What is the responsibility of the ED?
**What do your colleagues think about initiating buprenorphine/naloxone in the ED?**	- What are their successes?<br>- What are their concerns?
**What do you and your colleagues think about prescribing buprenorphine/naloxone for home initiation, or to-go?**	- What are your/their successes?<br>- What are your/their concerns?
**What are the key things to have in place in the ED for buprenorphine/naloxone initiation to occur successfully?**	- Special staff required?<br>- Medication availability?<br>- Follow up process?<br>- Staff training?
**What should happen in the ED for patients with an opioid use disorder in whom buprenorphine/naloxone has not worked in the past?**	- What would happen to this patient in your ED today?<br>- Should the ED initiate other forms of opioid agonist treatment?
**How many days of buprenorphine/naloxone should patients be given upon discharge from the ED? Should they receive a prescription?**	- Why this length of time?<br>- What do you think about giving someone a daily witnessed prescription for one week? For two weeks?
**Are you worried about the buprenorphine/naloxone that you give to patients being diverted to the illegal marker?**	- What are the risks and/or benefits of this?

	- Does the risk of diversion affect the length of your prescribing?

Topic Area III: Other treatments for patients with opioid use disorders in the ED

Questions:	Possible probes:
**How do you incorporate harm reduction discussions into your ED care, if at all?**	- Specific examples / incidents<br>- Do you talk to all your patients with opioid use about harm reduction?<br>- Does your incorporation of harm reduction strategies differ between patients?<br>- Does the level of harm reduction knowledge differ between physicians?
**Some patients will use substances while in the ED waiting room or while in the department. How do you think this should be managed in the ED?**	- Have you ever had an ED patient have an unintentional overdose in the waiting room or in the department?<br>- What would make this situation safer for patients and staff?
**What, if any, harm reduction services should be made available through the ED?**	- Naloxone kits? Why or Why not?<br>- Sterile Syringes? Why or Why not?<br>- Supervised consumption services? Why or Why not?<br>- Peer Support? Why or Why not?

**Naloxone Kit: Do you offer patients with opioid use disorders a naloxone kit in the ED?**	- Why or why not?<br>- How often do you give kits out?
**Sterile Syringes: Have you ever provided sterile syringes to patients who inject drugs?**<br>**[If yes] How often have you provided sterile syringes?**<br>**[If no] What are some of the reasons you haven't provided sterile syringes?**	- What was your experience?<br>- Does this happen routinely / ever in your ED?<br>- Does your hospital distribute sterile supplies?<br>- Have patients asked for sterile syringes?
**SCS: Do you refer patients to supervised consumption services?**	- Why or why not?<br>- How often do you refer patients?
**SCS: Do you think patients in the ED or the waiting room should have access to a supervised consumption service?**<br>**[If yes] Why do you think this is a good idea?**<br>**[If no] Why do you think this is a bad idea?**	- What would it take to make this happen at your hospital?
**Peer Support: Peer support workers are individuals with lived experience of substance use. Do you think there is a role for peer support workers in the ED?**	- Why or why not?<br>- How could they be most helpful in the ED?<br>- Do you have access to peer support workers in your ED?<br>- Do you refer patients to peer support workers in the community?

**I'd like you to imagine a scenario:**<br>**You are working a shift tomorrow and you are able to provide better care to a patient who injects opioids. What makes it better? (In other words, what can be immediately done to improve service delivery to this population)?**	- What would make it better in the short term? In the long term?<br>- What do you wish for in the future?<br>○ For yourself?<br>○ For your team?<br>○ For your hospital?
**Is there anything else you think we should be doing in the emergency department for patients with opioid use disorders?**	
**Can we contact you again if we need clarification on any of the responses you've shared with us today?**	- Email, phone contact information
